# Holistic approaches to trauma rehabilitation for forced migrants: assessing sleep as an associated factor for mental health, pain, and cardiovascular health

**DOI:** 10.1016/j.jmh.2026.100418

**Published:** 2026-05-29

**Authors:** Jennifer J. Esala, Sarah Lawrence, Cynthia J. Price, Jennifer Vanderminden, Bridget B. Gehrz, Sean Drummond, Ida Tchuisseu Fonkoue

**Affiliations:** a Center for Victims of Torture, 2356 University Ave W, St. Paul, MN 55114, USA; b University of Washington School of Nursing, Box 357266, Seattle, WA 98195, USA; c Think Bucket Consulting, 41 Plank Rd. Porter Corners, NY 12859, USA; d School of Psychological Sciences and Turner Institute for Brain and Mental Health, Monash University, 18 Innovation Walk Clayton, Victoria, 3800, Australia; e Divisions of Physical Therapy and Rehabilitation Science, Department of Family Medicine and Community Health, University of Minnesota, MN, USA

**Keywords:** Sleep quality, Forced migrants, Refugees, Mental health, Chronic pain, Cardiovascular health

## Abstract

**Introduction:**

Forced migrants experience trauma that adversely affects their health, leading to disproportionately high rates of sleep disturbance. Despite this burden, gaps remain in sleep research, clinical training, and the development of treatment protocols for this population. Likewise, limited research exists on the bidirectional relationship between sleep and other prevalent conditions in this population. This study assessed the relationship of sleep quality with anxiety, depression, post-traumatic stress disorder (PTSD), chronic pain, blood pressure, resting heart rate, and heart rate variability in a forced migrant population. It also reports on associations of sleep quality with trauma exposure and social participation.

**Materials and Methods:**

This was a cross-sectional study of 65 forced migrants who have at least mild chronic pain. Partial correlations were conducted with sleep quality and each outcome, controlling for age, body mass index (BMI), sex, length of time in the United States, and length of services at two trauma rehabilitation clinics.

**Results:**

Participants experienced high rates of poor sleep (92.3%). Sleep quality scores were significantly associated with PTSD, depression, anxiety, and chronic pain but not blood pressure, resting heart rate, or heart rate variability. Trauma exposure was significantly associated with poorer sleep quality, whereas social participation was associated with better sleep quality.

**Conclusion:**

Sleep quality was significantly associated with PTSD, anxiety, depression, and chronic pain in forced migrants and may represent an important clinical target in trauma rehabilitation. The contribution of this study is documenting these associations within a forced migrant population, where sleep remains understudied. Future work should develop and test sleep interventions adapted for the needs and context of forced migrants.

## Highlights

• Forced migrants had exceptionally high rates of poor sleep quality and short sleep duration.
• Poor sleep quality was positively associated with PTSD, depression, anxiety, and chronic pain.
• There was no association between sleep quality and cardiovascular health, which may be due to a lack of variability and small sample size.
• Migration-related trauma exposure was significantly associated with poorer sleep quality.
• Better sleep quality was significantly associated with greater social participation.

## 1 Introduction

Forced migrants experience migration-related traumas, stressors, and lifestyle changes that impact health outcomes. Before departure from their home countries, forced migrants are often exposed to violence, torture, physical injuries, witnessing the harm of loved ones, and living in conflict (Foster, 2001; Li, 2016; Miller and Rasmussen, 2010). During transit, they frequently encounter stressors such as lack of basic necessities (e.g., food, water, and medicine) and unsafe, unfamiliar conditions (Dow, 2011; Foster, 2001). After arrival in host countries, they face legal uncertainty, limited employment access, unfamiliar medical systems, racism, and lack of social support (Foster, 2001; Kim, 2016; Li, 2016; Miller and Rasmussen, 2010). Additionally, they experience significant lifestyle and dietary changes, often adopting poorer nutritional habits and more sedentary lifestyles, a common finding particularly when populations have limited resources (Akresh, 2007; Fennelly, 2007; Himmelgreen et al., 2007). For these reasons, forced migrants experience sleep disturbances at exceptionally high rates, with estimates ranging from 39% to 99%, far exceeding rates reported in general population studies (Aernout et al., 2021; Kerkhof, 2017; Richter et al., 2020).

Insomnia, defined as difficulty initiating or maintaining sleep, is highly prevalent among forced migrants, estimated at 32.6% to 73.3% (Baskaran et al., 2023; Jou and Pace-Schott, 2022; Lies et al., 2021; Richter et al., 2020). Objective sleep measures corroborate that sleep is highly disrupted among trauma-affected forced migrants (Ansbjerg et al., 2023). Sleep disturbances have serious downstream health consequences. Research has found inadequate or poor-quality sleep to be associated with a wide range of negative health outcomes, contributing to seven of the fifteen leading causes of death in the United States. In non-migrant populations, the data are clear: sleep disturbances are associated with higher rates of mental health disorders (Jou and Pace-Schott, 2022; Z. Li et al., 2025; Medic et al., 2017; Sutton, 2014), chronic pain (Alsaadi et al., 2014; Seow et al., 2020), cardiovascular disease (Covassin and Singh, 2016; Makarem et al., 2022; Wang et al., 2012), diabetes (Chattu et al., 2018; Kochanek et al., 2014), and cancer (Chattu et al., 2018; Kochanek et al., 2014), and meta-analyses show that sleeping less than seven hours per night increases mortality risk by 6–15% (Shah et al., 2025).

Despite the high prevalence of sleep disturbances among forced migrant populations and subsequent health consequences, there is a significant gap in available sleep research, clinical training, and treatment protocols for this population. For example, far more studies have examined mental health among forced migrants than have studied sleep health (Baskaran et al., 2023), despite sleep problems being equally or more common in this population and representing a hallmark symptom of post-traumatic stress disorder (PTSD) (Germain, 2013; Maher et al., 2006; Nappi et al., 2012; Schoenfeld et al., 2012; Spoormaker and Montgomery, 2008) and one that often persists even after PTSD treatment (Jou and Pace-Schott, 2022). There is a particularly large gap in understanding how sleep difficulties relate to other chronic health conditions experienced by forced migrants.

In sum, forced migrants face a high burden of chronic conditions that are often exacerbated by poor sleep. Although associations between sleep disturbance and mental health symptoms, chronic pain, and cardiovascular risk are well established in general populations, these relationships have been less frequently examined in forced migrant populations. This study addresses that gap by examining whether these established associations are also observed in a forced migrant sample, while additionally exploring links with trauma exposure and social participation.

## 2 Materials and methods

This was a cross-sectional study to examine sleep quality and related outcomes among a sample of 65 forced migrants with chronic pain who experienced torture and/or conflict-related trauma. The study data were drawn from a randomized controlled trial evaluating the effectiveness of massage therapy combined with somatic education for this population. Ethical approval for the study was obtained from the University of Minnesota Institutional Review Board (IRB ID: STUDY00021389).

### 2.1 Participant recruitment and eligibility

Study participants with chronic pain were recruited from two trauma rehabilitation clinics in St. Paul, MN that serve large, diverse populations of forced migrants. All potential participants were invited to meet with a research coordinator to review study procedures and provide informed consent. Clients were screened for eligibility and included if they reported Grade I or higher chronic pain, indicating a minimum of mild chronic pain. Eligible participants then completed a baseline assessment, the results of which are reported in this manuscript. Data was collected between May 2024 and December 2025.

### 2.2 Procedures

Following the initial screening, participants completed an in-person baseline assessment. Demographic and health-related data were collected including weight, height, and current pain and sleep medications. Trauma exposure, social participation, and immigration data were collected through clinical assessment and documentation. Language interpreters supported measures administration when the participant’s primary language was not English or the participant requested an interpreter. Due to literacy barriers, measures were administered verbally by a research coordinator rather than self-administered. All participants completed the Pittsburgh Sleep Quality Index (PSQI), Graded Chronic Pain Scale-Revised (GCPS-R), Hopkins Symptom Checklist-25 (HSCL-25), an adapted Posttraumatic Diagnostic Scale for DSM-5 (PDS-5), the Social Circumstances and Functioning Inventory, and seated blood pressure and heart rate variability.

### 2.3 Measures

*Pittsburgh Sleep Quality Index*

The Pittsburgh Sleep Quality Index (PSQI) is a validated, self-report measure of sleep quality over the previous month (Buysse et al., 1989). It includes 19 items that generate seven component scores: subjective sleep quality, sleep latency, sleep duration, sleep efficiency, sleep disturbances, use of sleep medication, and daytime dysfunction. Component scores are summed to produce a Global PSQI score from 0 to 21, with higher scores suggesting poorer sleep quality. A Global PSQI score greater than 5 indicates poor sleep. In this sample, the PSQI demonstrated good internal consistency (α = 0.80).

*Hopkins Symptom Checklist-25*

The Hopkins Symptom Checklist-25 (HSCL-25) is a validated, self-report 25-item inventory that assesses anxiety and depression symptoms (Derogatis et al., 1974). It includes a 10-item anxiety subscale and 15-item depression subscale, using a 4-point Likert scale, with higher scores suggesting greater symptom severity. Mean scores are calculated for each subscale with established cutoffs of ≥1.75 commonly used to indicate clinically significant symptoms. In this sample, the HSCL-25 demonstrated good internal consistency (anxiety α = 0.81 and depression α = 0.90).

*Posttraumatic Diagnostic Scale*

The Posttraumatic Diagnostic Scale (PDS-5) is a validated 20-item self-report measure that assesses PTSD symptoms in adults, based on DSM-5 diagnostic criteria (Foa et al., 2016). For this study, the original 5-point Likert response format was adapted to a simplified 4-point scale to facilitate administration with the clinic population. Total scores are calculated by summing responses across all 20 items, with higher scores indicating greater symptom severity. Due to the clinical adaptation and for comparability with prior literature, we calculated an approximate original-metric total by rescaling item scores (multiplying by 4/3) to recover the 0 to 4 item range (scale totaling 0–80). This rescaling facilitates descriptive interpretation but does not establish measurement equivalence with the validated PDS-5. Because the adapted response format has not been independently validated, scores may differ from those obtained using the original response scale in terms of psychometric properties and comparability with established clinical thresholds. Findings involving the adapted version of the PDS-5 should therefore be interpreted cautiously. In this sample, the PDS-5 demonstrated excellent internal consistency (α = 0.90).

*Graded Chronic Pain Scale-Revised*

The Graded Chronic Pain Scale-Revised (GCPS-R) is a validated, self-report measure that classifies chronic pain severity into four grades based on pain intensity and interference with daily activities: Grade I (low disability, low intensity), Grade II (low disability, high intensity), Grade III (high disability, moderately limiting), and Grade IV (high disability, severely limiting) (Von Korff et al., 2020). The GCPS-R allows for the calculation of the Pain, Enjoyment of Life and General Activity (PEG) Scale, which assesses pain intensity and its impact on general activity and enjoyment of life. The PEG consists of three items rated on a 0–10 scale; the total score is the average of these ratings, with higher scores indicating greater pain severity and interference. In this sample, the GCPS-R demonstrated acceptable internal consistency (α = 0.77).

*Social Circumstances and Functioning Inventory*

Social functioning was assessed with a 37-item standardized instrument validated with refugees. The constructs assessed with this inventory are basic needs, stabilization, employment, social support, adjustment, and community engagement (Vukovich and Tracey, 2017). These analyses focused on community engagement items.

*Seated Blood Pressure*

Seated blood pressure was measured using an automated oscillometric monitor (OMRON Upper Arm Monitor). Participants were seated in a resting position for 10 to 15 min before the first reading. An appropriately sized cuff was placed on the bare upper arm at heart level, and the arm was supported on a solid surface with legs uncrossed and feet flat on the ground throughout measurement. Participants refrained from talking during measurements. Three readings were obtained at sixty-second intervals to allow physiological stabilization, and if a reading failed, it was repeated after three minutes. The mean of the readings was used for analysis.

*Heart rate variability*

Heart rate variability (HRV) was measured using the Equivital wireless physiological monitoring system. HRV reflects the variation in time intervals between consecutive heartbeats and is a noninvasive indicator of autonomic nervous system function. Data were collected while participants were seated quietly for a minimum of 10 min. The measure administrator received training and followed a standardized protocol for data collection. All HRV data were reviewed for quality by an author (IF), who has specific expertise in HRV data collection and analysis. In this manuscript, we report on High Frequency power nu (HFpower nu), High Frequency (HF), Low Freq High Freq Ratio (LFHF), Short-term HRV (SDRR ms), HRV (RMSSD ms), and Time Domain HRV (pRR50). HF, LFHF, and pRR50 were non- normally distributed, indicating a negative skew. HRV variables were assessed for normality and transformed where appropriate. HF was transformed using a square-root transformation, and LFHF was transformed using a square-root transformation. For pRR50, a Log10-plus-one transformation provided the closest approximation to normality among the transformations evaluated; however, the transformed distribution remained somewhat non-normal.

*Trauma Exposure*

Trauma exposure indicators were collected as part of the intake process at the clinic. The variables included: family separation (separation from spouse, children, parents, or siblings due to forced migration); primary torture survivor (experienced torture based on the United States definition of torture); secondary torture survivor (a family member or someone close to primary torture survivor who experiences significant harm because of the torture inflicted on the primary survivor); physical violence by authorities (e.g., police, prison guard); sexual assault survivor; enduring physical injury (physical harm due to violence that currently causes pain or distress); forced labor / human trafficking, lived in hiding, and family member(s) killed or disappeared.

### 2.4 Statistical analysis

Data files were created and analyzed using IBM SPSS Statistics for Windows, Version 31.0. Descriptive statistics were calculated for demographic, health, and trauma history measures. Prior to conducting correlation analyses, data were examined for accuracy, missing values, and outliers.

Pairwise correlations were conducted with all variables of interest in this study, and additional partial correlations were conducted with sleep quality and each outcome, controlling for age, body mass index (BMI), sex, length of time in the U.S., and length of services with the Center for Victims of Torture (CVT) with 1= one year or more with CVT and 0= less than one year. The analysis examined the HSCL-25 and PDS-5 in their entirety and with the sleep item (one item per scale) excluded. The results with and without the sleep items included in the scales were consistent. We present the scales with the sleep items to follow in the univariate descriptives and without sleep items in all pairwise and partial correlation analyses.

Given the number of hypothesis tests conducted for sleep quality outcomes, we applied a Bonferroni correction to control the family-wise error rate. Corrected alpha thresholds were calculated for each family of tests, yielding α = 0.0028 (0.05/18) and α = 0.0038 (0.05/13). Because this approach is conservative and may increase the risk of Type II error, particularly in a modest sample, we present Pearson’s r correlation coefficients, unadjusted p-values, and the corresponding corrected alpha thresholds. Given the sample size of 65 forced migrants and the high prevalence of sleep disturbance, additional multivariable analyses were not pursued due to limited statistical power and the risk of model overfitting.

## 3 Results

### 3.1 Demographics

Demographic data included self-reported sex (male/female) and was evenly split by sex (see Table 1). The average age in the sample was in the late thirties (mean = 39.5, median = 37). The sample was diverse in educational background. Forty percent were married. A majority indicated having at least one child (66.2%). The average BMI for the sample was just above 25 (mean = 27.6, median = 26.4).

**Table 1:** Demographics of study participants.

	n (%)
Sex	
Male	33 (50.8)
Female	32 (49.2)
Education	
None	5 (7.7)
Primary School	11 (16.9)
High school/secondary school coursework	11 (16.9)
High school/secondary school graduate	11 (16.9)
College/vocational coursework	5 (7.7)
College/vocational degree	18 (27.7)
Graduate professional training	2 (3.1)
English Language	
None	12 (18.5)
Difficulty	14 (21.5)
Some difficulty	8 (12.3)
Proficient	31 (47.7)
Marital Status	
Divorced	4 (6.2)
Married	26 (40)
Separated	4 (6.2)
Single	27 (41.5)
Widowed	4(6.2)
Total Number of Biological Children	
0	22 (33.8)
1	7(10.8)
2	13 (20)
3	12 (18.5)
4	5 (7.7)
5	4 (6.2)
6	2 (3.1)
Country of Origin	
Cameroon	22 (33.8)
Ethiopia	16 (24.6)
Togo	9 (13.8)
Myanmar	6 (9.2)
United Republic of Tanzania	2 (3.1)
Afghanistan	1 (1.5)
Cambodia	1 (1.5)
Gambia	1 (1.5)
Ghana	1 (1.5)
Guinea	1 (1.5)
Lao People’s Democratic Rep	1 (1.5)
Morocco	1 (1.5)
Nigeria	1 (1.5)
Sierra Leone	1 (1.5)
Somalia	1 (1.5)

About one-quarter had only primary school or no formal education; one-third attended or completed high school; and over one-quarter of the sample completed a college or vocational degree. Likewise, participants were diverse in English language proficiency: about half (47.7%) were proficient in English; one-third had difficulty speaking English (33.8%); and nearly one-fifth had no English proficiency (18.5%). One-third were from Cameroon (33.8%), one-quarter from Ethiopia (24.6%), followed by 9 participants from Togo (13.8%) and 6 participants from Myanmar (9.2%). Two participants were from the United Republic of Tanzania. The median time since arrival in the U.S. was 15 months (mean = 42.78), reflecting a single high outlier, which was trimmed for correlational analysis.

### 3.2 Trauma exposure

Study participants reported a high level of trauma exposure (see Table 2). Nearly all participants were primary survivors of torture (93.8%), meaning that they directly experienced torture. Over eighty percent were separated from at least one family member due to forced migration (81.5%), with over half of parents in the study sample being separated from children (72.1%). Eighty percent experienced physical assault by an official or authority, almost forty percent experienced sexual assault by an official or authority (38.5%), and sixty percent reported enduring physical harm since the assault experience. Fifty percent of the sample reported having a family member who was tortured (50.8%) or killed / disappeared (55.4%). Detainment lasted a median of 15 days, with a maximum value of 1825 days.

**Table 2:** Trauma history descriptive statistics.

		n (%)		
Current Family Separation		53 (81.5)		
Spouse Separation		13 (25.4)		
Child Separation (among those with children)				
0		12 (27.9)		
1		4 (9.3)		
2		11 (25.6)		
3		10 (23.3)		
4		2 (4.7)		
5		3 (7.0)		
6		1 (2.3)		
Survivor of Torture - Primary†		61 (93.8)		
Survivor of Torture - Secondary†		35 (53.8)		
Physical Violence by Authorities		52 (80.0)		
Survivor of Sexual Assault by an official or authority		25 (38.5)		
Enduring Physical Injury		39 (60.0)		
Forced Labor / Human Trafficking		10 (15.4)		
Lived in Hiding		9 (13.8)		
Family Members Killed or Disappeared		36 (55.4)		

	n	Mean (SD)	Median (IQR)	
Age at First Torture	56	28.84 (9.7)	29 (10)	
Number of Times Detained	62	1.9 (1.1)	2 (2)	
Longest period of Detainment in days	61	159.8 (345.0)	15 (171.5)	

† A primary torture survivor is a person who has directly experienced torture, while a secondary torture survivor is a family member or someone close to primary torture survivor who experiences significant harm because of the torture inflicted on the primary survivor.

### 3.3 Baseline sleep statistics

Based on the Global PSQI score (with a cutoff >5 indicating poor sleep), 92.3% (n = 60) of the study sample were classified as having poor sleep. In contrast, only 43.8% (n = 28) self-reported poor sleep in the PSQI self-report sleep quality item. The mean Global PSQI sleep quality score was 12.26 (Table 3). On average, study participants reported sleeping 4.6 h per night.

**Table 3:** Sleep quality descriptive statistics.

	N	Mean (SD)	Median (IQR)	
Sleep quality score	65	12.26 (4.1)	13 (5)	
Duration (hours)	65	4.61 (1.6)	4.5 (2.5)	
Latency (minutes)	64	87.78 (83.5)	60 (90)	
Efficiency (%)	65	68.88 (23.7)	70.59 (40.5)	

Sleep Disturbances	Not during the last month n (%)	Less than once a week n (%)	Once or twice a week n (%)	3+ times per week n (%)
Wakes up in the middle of the night	5 (7.7)	2 (3.1)	16 (24.6)	42 (64.6)
Wakes up in the middle of the night due to…				
Get up to use the bathroom	16 (24.6)	2 (3.1)	16 (24.6)	30 (46.2)
Cannot breathe comfortably	38 (58.5)	10 (15.4)	7 (10.8)	10 (15.4)
Cough or snore loudly	45 (69.2)	5 (7.7)	4 (6.2)	2 (3.1)
Feel too cold	40 (61.5)	6 (9.2)	9 (13.8)	10 (15.4)
Feel too hot	40 (61.5)	3 (4.6)	7 (10.8)	15 (23.1)
Had bad dreams	19 (29.2)	9 (13.8)	15 (23.1)	22 (33.8)
Have pain	10 (15.4)	8 (12.3)	14 (21.5)	33 (50.8)

Sleep latency refers to the time it takes to fall asleep. A latency of 30 min or less is generally considered normal. In this sample, the mean sleep latency was 87.78 min (median = 60) with a non-normal distribution. Based on the PSQI Sleep Latency component, 90.6% of participants were classified as having poor sleep latency.

Sleep efficiency refers to the percentage of time in bed sleeping, with higher percentages reflecting better efficiency. A sleep efficiency of ≥85% is generally considered normal. In this sample, the mean efficiency was 68.88%. Based on the PSQI Sleep Efficiency component, 55.38% of participants were classified as having poor sleep efficiency.

Sleep disturbance reflects the frequency of overnight disruptions such as waking, discomfort, or other problems. Sleep disturbances were common in this sample, a majority (64.6%) of the sample indicated waking up in the middle of the night three or more times per week. Over half of participants (50.8%) reported pain disturbing their sleep three or more times per week, and nearly half (46.9%) reported waking to use the bathroom three or more times per week. Difficulties with temperature regulation were also notable, 58.5% (n = 38) endorsed being too hot or too cold three or more times per week. Nearly one-third of participants (33.8%) reported frequent bad dreams, while 15.4% experienced breathing difficulties at least three times per week.

Daytime dysfunction, as measured by the PSQI, reflects the degree to which sleep problems interfere with daytime alertness and functioning. In this sample, 44.6% (n = 29) experienced some daytime dysfunction, 10.3% (n = 6) reported it three or more times per week.

Despite the high rates of short sleep, long sleep latency, and sleep disturbance, most participants (67.6%, n = 44) reported not using sleep medication. Overall, 32.3% (n = 20) reported some medication use, including 23.1% (n = 15) who used sleep medication three or more times per week.

In response to the open-ended question about sleep disturbance, participants described a range of additional reasons for disturbed sleep. Psychological factors were frequently mentioned, including worries or memories about past traumatic experiences, flashbacks, sadness or depression, and ongoing stress related to current circumstances or family members in their country of origin. Environmental disturbances such as noise (e.g., sirens, neighbors), lights, and the early timing of morning prayer were also reported. Social and household conditions contributed to sleep disruption, including unsafe or unstable housing, lack of housing, and disturbance from roommates, children, or infants. Other contributors included menopause symptoms, difficulty moving, hunger, and uncomfortable sleeping arrangements (e.g., insufficient bedding or an uncomfortable bed).

### 3.4 Baseline health outcome statistics

Overall mental health symptomology in the study sample was high. The average anxiety (2.08) and depression (2.57) scores were above the diagnostic cut point of 1.75 (Table 4). From the adapted PDS-5, the mean PTSD score was 39.8, which is above the cutoff score of 28 for identifying a probable PTSD diagnosis. Pain was also high in this study sample, with a mean PEG score of 6.45, which is above the threshold of PEG ≥4 used in research to identify patients requiring pain intervention (Chan et al., 2021). Mean systolic and diastolic blood pressure readings were non-hypertensive (120.56 / 78.41), and the mean resting heart rate was considered healthy (71.8), with optimal values being 60–80 bpm. HRV indicators appeared to be comparable to studies with other forced migrant populations (Slewa‐Younan et al., 2012).

**Table 4:** Health outcomes descriptive statistics.

	n	Mean (SD)	Median (IQR)	% Meeting Diagnostic Threshold
Anxiety	65	2.08 (0.66)	2.10 (1.00)	64.6
Depression	64	2.73 (0.70)	2.96 (1.10)	87.5
PTSD	59	41.31 (18.90)	44.00 (32.00)	74.6
Pain score	65	6.45 (2.30)	6.67 (3.00)	89.2
Systolic blood pressure	65	120.56 (16.70)	120.70(23.30)	40.0†
Diastolic blood pressure	65	78.41 (10.00)	75.33 (11.70)	
High Frequency (HFpower nu)	61	34.55 (13.78)	33.46 (22.50)	
High Frequency (HF)	62	19.53 (10.75)	16.22 (14.90)	
Low Freq High Freq Ratio (LFHF)	62	2.47 (1.63)	1.98 (2.20)	
Short-term HRV (SDRR ms)	62	50.90 (21.51)	43.84 (30.15)	
HRV (RMSSD ms)	62	36.68 (20.56)	31.59 (29.30)	
Time domain HRV (pRR50)	62	16.52 (17.05)	9.59 (31.39)	
Resting heart rate	62	71.80 (9.50)	71.08 (11.20)	

† Percent of sample classified as having hypertension with a systolic blood pressure >130 mmHg OR a diastolic blood pressure (DBP) >80 mmHg.

### 3.5 Associations between sleep and other health outcomes

There was a moderate positive correlation between sleep quality and depression (see Table 5), indicating that worse sleep (higher PSQI score) was correlated with greater symptoms of depression, including after the Bonferroni correction. There were also moderate positive correlations between sleep quality and PTSD, anxiety, and chronic pain. The correlation between sleep quality and PTSD remained statistically significant after the conservative Bonferroni correction with an *α of 0.05 (corrected to.0028) while* anxiety and chronic pain were significant at an α of 0.10 (corrected to 0.006). There were no significant correlations between sleep quality and blood pressure, resting heart rate, or HRV. Sleep quality was not associated with most of the control variables, including sex, age, and BMI. Poor sleep quality was associated with attending trauma rehabilitation services for a year or longer, and with the number of months since immigration.

**Table 5:** Pearson’s *r* Correlation of sleep quality (PSQI) and chronic health conditions and physiological measures (blood pressure, heart rate, HRV) with no controls^a^ and with controls^b^ (age, sex (female), BMI, CVT for one year or more, and months since immigration).

Measures	PSQI Score *R*	Sig		PSQI Score^b^ *r*	Sig
Anxiety	.302	.015		.396	.004
Depression	**.539**	**<0.001**		**.518**	**<0.001**
PTSD	**.415**	**.001**		**.479**	**<0.001**
Pain Score	**.398**	**.001**		.400	.004
Systolic blood pressure	.033	.796		−0.010	.946
Diastolic blood pressure	.173	.169		.103	.476
High Frequency (HFpower nu)	.068	.603		.038	.802
High Frequency (HF)	.074	.565		−0.013	.931
Low Freq High Freq Ratio (LFHF)	−0.031	.812		.035	.817
Short-term HRV (SDRR ms)	−0.079	.544		.030	.844
HRV (RMSSD ms)	−0.020	.875		.085	.569
Time Domain HRV (pRR50)	−0.041	.751		.111	.458
Average BPM	−0.03	.818		−0.101	.501
Female	.189	.133			
Age	.169	.179			
BMI	.136	.323			
CVT for 1 year or more	.246	.048			
Months since immigration	.364	.003			

Note: Bonferroni correction for α= 0.05/18= 0.0028, cells in bold remain statistically significant after correction.

### 3.6 Sleep quality and trauma exposure

**Fig. 1:**
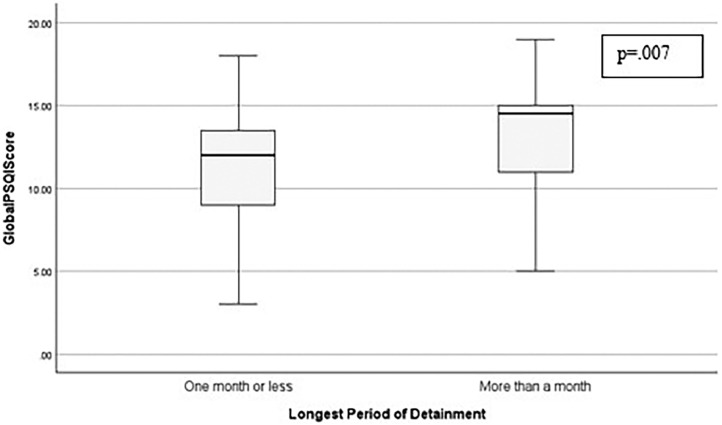
Sleep quality (Global PSQI) score by length of detainment.

**Fig. 2:**
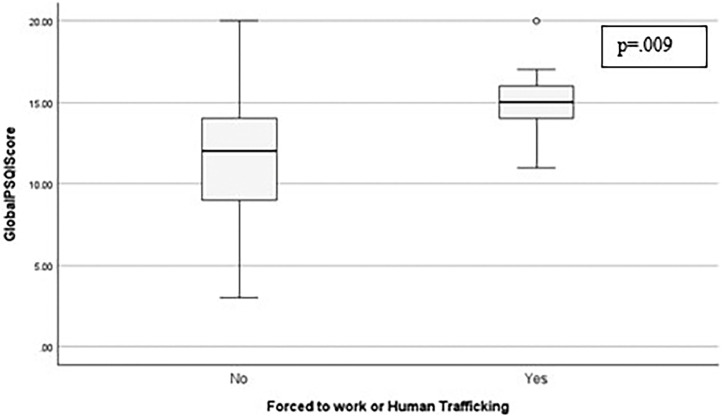
Sleep quality (Global PSQI) score by forced labor / human trafficking.

Participants who experienced forced labor or human trafficking showed significantly worse sleep (*r* = 0.322; *p* = .009), as did those who were detained for more than a month (*r* = 0.340; *p* = .007) (see Fig. 1, Fig. 2). Other trauma indicators were not significantly associated with sleep.

**Sleep Quality and Social Participation**

**Fig. 3:**
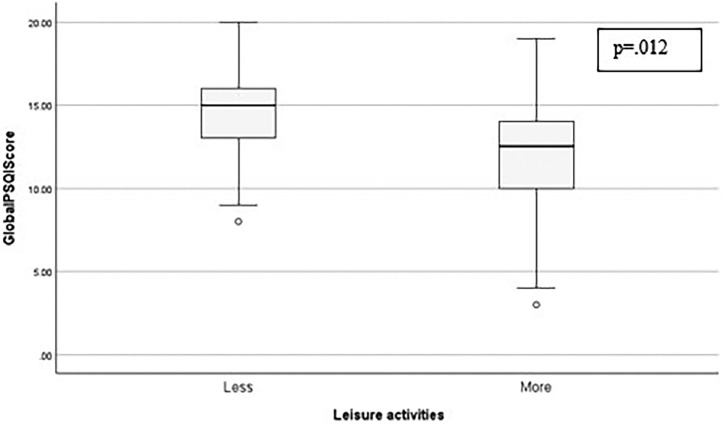
Sleep quality (Global PSQI) score by engagement in leisure activities.

**Fig. 4:**
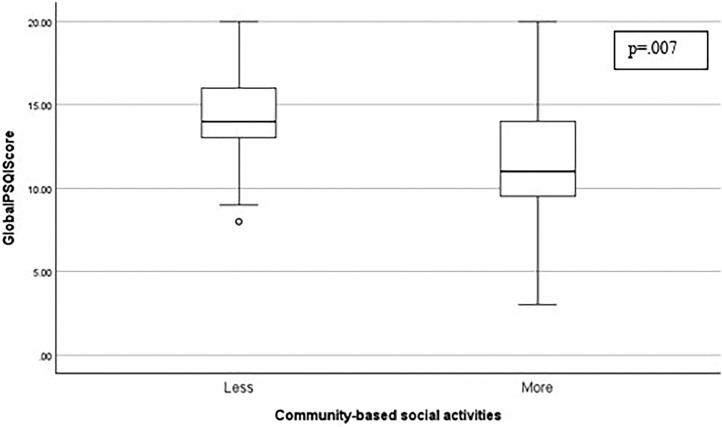
Sleep quality (Global PSQI) score by community-based social activities.

Subscales and selected items in the Social Circumstances and Functioning Inventory were examined in this analysis. The extent to which basic needs were met was not associated with sleep quality. Two items related to social participation were significantly and negatively associated with sleep quality (see Fig. 3, Fig. 4). Those who engaged in frequent leisure activities showed significantly better sleep quality (*r* = - 0.326; *p* = .012), as did those who reported participating in community-based social activities (*r* = - 0.353; *p* = .007). Other social participation indicators were not significantly associated with sleep.

## 4 Discussion

Sleep disturbance is a critical issue for forced migrants. This study found poor sleep quality and high rates of sleep disturbance in this forced migrant sample. Sleep quality was significantly associated with several chronic health conditions, including depression, anxiety, PTSD, and chronic pain, which is consistent with research in general populations. Sleep quality was not significantly associated with blood pressure, heart rate, or HRV, which is not consistent with research in general populations. This lack of association may be due to the limited variability in sleep quality in this population and the small sample size. Social participation and trauma exposure were both associated with sleep quality.

In this sample, the mean sleep duration was 4.61 h per night. Studies suggest that 5 h or less of sleep per night prompts an acute stress response and increased risk for chronic disease (Covassin and Singh, 2016; Shah et al., 2025). Sleeping <7 h per night increases mortality risk by 6–15% (Chaput et al., 2020; Shah et al., 2025). Only 7 of 65 participants were classified as having an ideal sleep duration. We identified significant associations of poor sleep quality with length of detention and experience of forced labor or human trafficking. Notably, these associations were identifiable in this sample despite high rates of sleep disturbance and trauma exposure both with minimal variability. Other research has shown that sleep mediates the association between premigration trauma exposure and current posttraumatic symptoms (Lies et al., 2021). This further suggests a therapeutically central role of sleep in trauma exposed populations. This study also found that >90% of participants met the criteria for poor sleep according to the Global PSQI score, but less than half self-reported poor sleep on the self-report PSQI sleep quality item. This discrepancy has important clinical implications, suggesting that sleep disturbances may be under identified in clinic settings and that sleep screeners may be needed in clinical practice to ensure sleep disturbances are adequately identified and treated in forced migrant populations.

Poor sleep quality was associated with attending trauma rehabilitation services for a year or longer. The observed correlation between treatment duration and poor sleep quality may reflect the underlying severity of sleep disturbance, related mental health symptomology, and the subsequent need for longer treatment, not a causal effect of treatment.

This study demonstrated that the well-established associations between sleep quality and mental health symptoms in general populations are also present in a forced migrant population with chronic pain and trauma exposure. Research shows that psychological disorders negatively impact sleep, while poor sleep contributes to the development and persistence of mental health symptoms (Jou and Pace-Schott, 2022; Li et al., 2025; Medic et al., 2017; Sutton, 2014). For instance, sleep disturbance is a cardinal symptom of major depressive disorder (Freeman et al., 2020; Sutton, 2014), yet sleep problems also precede and exacerbate depression rather than only resulting from it (Freeman et al., 2020; Scott et al., 2021). Chronic insomnia predicts at least a twofold increase in the likelihood of subsequent depression (Baglioni et al., 2011; Hertenstein et al., 2019) and a fivefold increase for at least one psychiatric diagnosis (Sarsour et al., 2010; Sutton, 2014). Similarly, sleep difficulties prior to or soon after traumatic events predict the later onset of PTSD (Freeman et al., 2020; Gehrman et al., 2013; Koffel et al., 2016). A meta-analysis of 65 randomized control trials (Scott et al., 2021) and a subsequent synthesis (Li et al., 2025) both confirmed that improvements in sleep quality produce reductions in depression, anxiety, and overall psychological distress, with a dose-response relationship. These broader findings in combination with correlational findings from the present study suggest that sleep may be a promising area for intervention research for improved mental health for forced migrants.

Research, including a meta-analysis of a large community sample (N = 33,595), shows a U-shaped relationship between sleep duration and depression. Sleeping seven to eight hours was associated with the lowest risk of depression and that risk increased with less sleep (Kaneita et al., 2006; Li et al., 2025, 2023; Wang et al., 2016). Existing research on sleep duration among forced migrants focuses primarily on sleep disturbances and sleep quality rather than reporting on sleep duration categories (long and short sleep). In this sample, only three percent of clients had long sleep (both at just 9 h), whereas 89.2% had short sleep (<7 h). The prevalence of long sleep in forced migrant populations, and its association with depression, is unknown. The high rates of PTSD, poor sleep conditions, and social circumstances make it less likely that forced migrants will present with long sleep. This has potential implications for sleep treatment and related treatment of depression in this population. Moreover, sleep treatment, as compared to psychotherapy, is often a more culturally acceptable treatment target, which may positively impact access, retention, and thus depression symptom reduction.

A critical aspect of trauma and torture rehabilitation is social participation. The United Nations Convention Against Torture (UNCAT) identifies the full inclusion and participation in society as a component of full rehabilitation (“Convention against Torture and Other Cruel, Inhuman or Degrading Treatment or Punishment,” 1984). Research in general populations suggests that sleep improvements may support enhanced social participation among forced migrants (Dumser et al., 2025; Jespersen and Vuust, 2012). In this study, we observed an association of sleep quality with community engagement and leisure activities. It is possible that better sleep supports greater social participation due to improvements in energy, mood, and overall psychological and physical functioning. It is also possible that greater social participation improves sleep via increased physical activity and improved mood states. The association holds in research with general populations with better sleep associated specifically with higher social functioning, social participation, and less loneliness (Hammer and Bonfils, 2024; Lallukka et al., 2018). Previous research with forced migrants with PTSD did not show sleep problems to be associated with social impairment or quality of life, but they did not address the relationship between sleep disturbance and specific forms of positive social participation (Schumm et al., 2023).

The present study found that the well-documented association between sleep quality and chronic pain in general populations was also evident in this forced migrant sample. Despite the high prevalence of both chronic pain and sleep disturbance in forced migrant populations, there remains a limited amount of research examining the relationship between these conditions (Baskaran et al., 2023). One study with forced migrants showed that sleep quality was significantly associated with pain intensity and interference, and it mediated the effect of PTSD severity on pain interference (Friis et al., 2025). In research with general populations, the association between sleep disturbance and pain has been shown to be complex and bidirectional, involving potential shared mechanisms such as hyperarousal, sympathetic nervous system activation, hormonal changes, and inflammation. Poor sleep contributes to increased pain intensity, while heightened pain disrupts sleep (Alsaadi et al., 2014). Both short sleep and poor sleep quality are independently associated with chronic pain (Seow et al., 2020). Interventions aimed at improving sleep quality have been shown to contribute to reductions in chronic pain (Selvanathan et al., 2021). Pain is notoriously difficult to treat among forced migrants and survivors of torture populations, who commonly present with co-occurring physical and psychological conditions that often go untreated for extended periods of time (Lies et al., 2019; Lurie et al., 2024). In general populations, there is preliminary evidence that sleep interventions, most predominantly CBT-Insomnia, can be used to treat pain (Ho et al., 2019; Koffel et al., 2016; Whale et al., 2022). Proposed mechanisms include the role of sleep in restoring endogenous pain inhibition, reducing systemic inflammation, and improving maladaptive behaviors and thought patterns. Sleep treatment may represent an effective, noninvasive, and highly accessible treatment to support pain management for forced migrant populations but requires further investigation.

There is currently no research beyond this study that examines the relationship between sleep and cardiovascular health indicators among forced migrant populations. This study found no relationship between sleep quality or duration with blood pressure, heart rate, or HRV, which may be due to the lack of variability in sleep quality in this sample and the small sample size. Nonsignificant findings should be interpreted as preliminary and considered in light of the sample’s high overall level of sleep disturbance, modest sample size, and limited statistical power. There is robust literature in general populations on the strong association between sleep and cardiovascular health indicators. Research in general populations shows an association between short sleep and poor sleep quality with various types of heightened cardiovascular risk, such as hypertension, coronary heart disease (CHD), and stroke (Covassin and Singh, 2016; Makarem et al., 2022; Wang et al., 2012). Short sleepers have heightened risk of fatal and non-fatal CHD events (Covassin and Singh, 2016; Jackson et al., 2015; Makarem et al., 2022). The risk of CHD increases by 11% for every one-hour decrease in sleep below the recommended range (Wang et al., 2016). Individuals experiencing insufficient sleep are also 1.2 to 1.61 times more likely to develop hypertension (Shah et al., 2025). The present study sample did not reflect the broader population and begs further investigation.

This sample had a mean non-hypertensive blood pressure, despite their high rates of trauma exposure, mental health symptomology, chronic pain, and sleep disturbance, all of which indicate autonomic nervous system dysfunction. Another study found that 30% of adult forced migrant patients in Canada (n = 1063) had elevated blood pressure (Redditt et al., 2015) and the present study showed 40% had high blood pressure. The Centers for Disease Control and Prevention (CDC) reports, however, that about 50% of the U.S. population have high blood pressure (CDC, 2023). This suggests that these forced migrant samples had a greater percentage with normal blood pressure than host country populations. A part of this finding could be accounted for by the healthy immigrant effect (HIE), which is the epidemiological finding that immigrants to high-income countries show better health profiles (including cardiovascular health) than the native-born populations in high-income host countries (Markides and Rote, 2019; Okrainec et al., 2015; Vyas et al., 2024). While a population-based study found that the HIE did not hold for forced migrants as a protective factor against cardiovascular risk, studies did not report specifically on hypertension statistics (Okrainec et al., 2015; Tu et al., 2015). Immigrant sub-groups face different required health screenings and experience distinct levels of trauma exposure prior to and after arrival, which are some potential explanations for why HIE presents differently in immigrant sub-groups.

This study suggests several areas for future research. Sleep disturbance may be under-identified in forced migrant populations, and there is a need to design and test sleep screeners adapted for the life conditions and histories of forced migrants to ensure sleep disturbances are adequately identified and treated. Additionally, treating sleep may be a promising approach to support mental health treatment for forced migrants; future research should assess the efficacy and explore the implementation of sleep interventions to treat both mental health and sleep disturbances in forced migrant populations. Likewise, sleep treatments may be effective, noninvasive, and highly accessible for pain management among forced migrant populations, which warrants additional study. This study showed no correlation between sleep and cardiovascular health indicators, which runs counter to research in general populations. Future research should explore the association between sleep and cardiovascular health and the distinct way the HIE may present in forced migrant sub-populations.

There are limitations to this study. The sample is not generalizable to the forced migrant population. The sample is small, treatment seeking, and only included participants with chronic pain. Due to these limitations and the anticipated characteristics of the broader forced migrant population, there is little variation in the sleep quality, which tends to be very poor. There are high levels of trauma exposure in this sample and, in some cases, little variation in related variables, such as the torture status. Unmeasured factors, including trauma severity, medication use, comorbid health conditions, and other clinical or social factors, may have influenced the observed associations. Lastly, there is not global representation in the study sample, with disproportionate representation from Cameroon and Ethiopia.

## 5 Conclusion

This study showed sleep quality to be exceptionally low in a sample of forced migrants and that sleep quality was associated with PTSD, anxiety, depression, and chronic pain. These associations are consistent with prior literature, but this study extends that evidence to a forced migrant population. Sleep disturbances are highly prevalent and clinically relevant for forced migrants, while also not well addressed in trauma rehabilitation for this population. To fully meet the task of trauma rehabilitation for forced migrants, it is essential to treat sleep disturbances and assess whether treating sleep improves associated chronic health conditions.

## CRediT authorship contribution statement

**Jennifer J. Esala:** Writing – review & editing, Writing – original draft, Supervision, Software, Resources, Project administration, Methodology, Investigation, Funding acquisition, Formal analysis, Data curation, Conceptualization. **Sarah Lawrence:** Writing – review & editing, Methodology, Investigation, Data curation. **Cynthia J. Price:** Writing – review & editing, Resources, Investigation, Conceptualization. **Jennifer Vanderminden:** Writing – review & editing, Writing – original draft, Formal analysis. **Bridget B. Gehrz:** Writing – review & editing, Project administration, Investigation, Data curation. **Sean Drummond:** Writing – review & editing, Methodology. **Ida Tchuisseu Fonkoue:** Writing – review & editing, Resources, Methodology, Formal analysis, Data curation, Conceptualization.

## Declaration of competing interest

The authors declare the following financial interests/personal relationships which may be considered as potential competing interests: Jennifer J Esala reports financial support was provided by Massage Therapy Foundation. If there are other authors, they declare that they have no known competing financial interests or personal relationships that could have appeared to influence the work reported in this paper.
